# Intestinal Donors in Early Infancy: Are Immune-mediated Complications More Problematic?

**DOI:** 10.1097/TXD.0000000000001879

**Published:** 2025-11-14

**Authors:** Angus Hann, Girish Gupte, Jane Hartley, Rebecca Jeyaraj, Evelyn Ong, M Thamara P.R. Perera, Darius F. Mirza, Khalid Sharif

**Affiliations:** 1 Liver and Intestinal Transplant Unit, Birmingham Children’s Hospital, Birmingham, United Kingdom.; 2 Liver Unit, Queen Elizabeth Hospital, Birmingham, United Kingdom.; 3 Centre for Liver and Gastrointestinal Research, Institute of Immunology and Immunotherapy, University of Birmingham, Birmingham, United Kingdom.

## Abstract

**Background.:**

Small children awaiting intestinal transplantation are disadvantaged because of a lack of size-compatible donors. To achieve suitable access to transplantation, using intestinal grafts from very young donors is an option. However, there is a lack of literature describing the posttransplant immunological complications associated with these grafts. Our aim was to report the outcome of intestine-containing grafts from very young donors (6 mo and younger).

**Methods.:**

A single-center retrospective study of all children who received an intestine-containing allograft between January 1993 and December 2020. To investigate the effect of donor age, the participants were separated into 3 groups. These comprised early infancy (EI, donors 6 mo and younger), late infancy (LI, 7–12 mo), and an early childhood (EC, 13–36 mo). The primary outcome was clinically significant immunological complications, defined as severe rejection and/or graft versus host disease.

**Results.:**

During the study period, 105 pediatric intestinal transplants were performed and 35 were from donors aged 36 mo and younger. The EI, LI, and EC donor groups comprised 5, 9, and 21 patients, respectively. The indications for transplant, recipient age (in months) (EI: 17 [15–20], LI: 18 [11–30], EC: 24 [15–47]), and maintenance immunosuppression were similar between groups. Significant immunological complications occurred more frequently in the EI group (EI: 4/5 [80%], LI: 5/9 [56%] EC: 7/21 [33%], *P = *0.134), and 3 of 5 of the recipients died as a direct result of these complications.

**Conclusions.:**

The findings suggest that immunological complications are more frequent with grafts from donors within the first 6 mo of life and caution should be applied with their use.

## INTRODUCTION

Intestinal transplantation (IT) is a lifesaving treatment for individuals who have lost nutritional autonomy due to intestinal failure and cannot be safely managed with parenteral nutrition or other surgical intervention.^1-3^ Children with irreversible intestinal failure and progressive intestinal failure-associated liver disease, especially within the first few years of life, are at a disadvantage due to the scarcity of appropriately size matched grafts available in the United Kingdom. This may result in excessive wait times and put the patient at risk of increased morbidity and mortality.^4,5^ The median age of pediatric IT recipients in the United Kingdom is 3 y of age; therefore, the space within the abdominal cavity does not have the capacity to fit a small intestine or multivisceral graft from an adult donor.^6^ Various innovative approaches have been implemented at our center (liver and/or intestinal reduction and staged abdominal closure) to improve the transplantation prospects of small children.^4,7^ To avoid a patient missing their therapeutic window for IT, using donors in EI has been another strategy used. A similar approach has been used at other centers, with a noted reduction in time to transplantation.^8^

The determination of death by neurological criteria in the United Kingdom was revised in 2015 to include infants younger than 2 mo,^9^ and recommendations exist that infants aged older than 2 mo are assessed using the same criteria as adults.^10^ Therefore, the opportunity to use abdominal organs from these very young donors (younger than 6 mo) has increased. A recent single-center report from the United States reported a higher technical complication rate with intestine-containing grafts from donors aged younger than 1 y. However, when controlled for recipient age, a higher graft survival was observed in recipients of grafts from donors younger than 1 y. This study did not report specific outcomes for the subgroup of neonates or very young infant donors, a group with which we have concerns regarding the severity of immune-mediated sequalae.^8^

The impact donor age has on pediatric IT outcomes is poorly reported in the literature.^8^ More specifically, the outcomes of grafts from very young donors need further investigation. Successful outcomes from neonatal donors have been described in the form of case reports only, which are subject to significant publication bias. A child’s intestine is undergoing a complex process of maturation during the first year of life and is exposed to a variety of different dietary antigens throughout this period. In addition, the phenotype of passenger immune cells within the graft from a very young donor may differ from that of an older individual, and the graft-recipient interaction may have different clinical manifestations. These factors may impact the immunogenicity of the intestine-containing graft, and the associated risks need to be balanced against the benefits of these donors, which are seemingly of ideal size for recipients with small abdominal domains. The aim of this study is to report on IT recipients who received grafts from donors in early infancy (EI; younger than 6 mo) and to compare the outcome with grafts from donors in late infancy (LI) and early childhood (EC).

## MATERIALS AND METHODS

All recipients of an intestine-containing allograft at our institution between January 1993 and December 2020 were considered for inclusion in this retrospective, observational cohort study. Institutional review board approval was not required for this retrospective study; however, established policies regarding data protection were followed. Our IT database was reviewed to determine the age of the organ donor, and patients were excluded if the organ donor was older than 36 mo. The included patients were divided into 3 groups according to donor age. These comprised an EI donor group (6 mo and younger), a LI group (7–12 mo), and an EC group (13–36 mo). Demographic, donor, surgical, and postoperative characteristics were collected from hospital medical records. Indications for IT were classified as short bowel syndrome, dysmotility, or a mucosal disorder. The primary outcome was the incidence of significant immune-mediated complications, and these were defined as severe rejection (acute or chronic) or graft versus host disease (GVHD). Secondary outcomes included 1-, 3-, and 5-y graft survival; the reason for graft loss or death; and the incidence of posttransplant lymphoproliferative disorder (PTLD). The diagnostic criteria for severe acute rejection were consistent with those previously published^11^ and required mucosal erosion or ulceration with a significant inflammatory infiltrate. All patients were managed according to the routine postoperative care of that time period. The postoperative immunosuppressive regimen has remained the same since 2002, comprising an interleukin-2 receptor antagonist, tacrolimus, and steroids as the standard treatment. An antimetabolite agent was included for patients identified as being at higher risk of rejection. Our approach and the changes during recent decades have been previously described in detail.^12^

Statistical analysis was performed via SPSS (version 25.0, IBM Corp, Armonk, NY). Continuous variables are reported as medians with interquartile range. Categorical variables were assessed with the Pearson chi-square test for independence. Continuous variables were compared with the Kruskal-Wallis test. An alpha value of 0.5 was considered the threshold for statistical significance.

## RESULTS

During the study period, 105 IT procedures were performed and our institution and 35 were included in this study cohort because the donor was aged 36 mo and younger. Grouping based on donor age resulted in 5, 9, and 21 patients being in the EI, LI, and EC groups, respectively. Donor and recipient characteristics are displayed in Table 1. Despite differences in donor age and weight, the age (in months) of the recipients in each group was similar (*P = *0.29). The donor-to-recipient age ratio was significantly lower in the EI group in comparison with both the LI and EC groups (EI: 0.14 [0.12–0.25], LI: 0.66 [0.38–0.87], EC: 1.04 [0.59–2.11], *P* < 0.01). The donor-to-recipient weight ratio was lower in the EI group than both the LI and EC groups; however, the difference was not statistically significant. Indications for IT were similar between groups (Table 1).

**TABLE 1.** Characteristics of recipients and donors

	EI (≤6 mo) N = 5	LI (7–12 mo) N = 9	EC (13–36 mo) N = 21	*P*
Female recipient	5 (100%)	6 (67%)	9 (43%)	0.054
Waitlist duration, d, median (IQR)	259 (178–322)	259 (41–415)	132 (63–309)	0.569
Indication				0.09
Short bowel	2 (40%)	4 (44%)	15 (71%)	
Dysmotility^*a*^	2 (40%)	2 (22%)	6 (29%)	
Mucosal disorders	1 (20%)	3 (34%)		
Premature	4 (80%)	7 (78%)	6 (29%)	0.004
Preoperative PN only	3 (60%)	1 (11%)	5 (24%)	0.354
Recipient age, mo (range)	17 (15–20)	18 (11–30)	24(15–47)	0.29
Donor age, mo (range)	2 (2–5)	12 (11–12)	24 (24–36)	**<0.001**
Donor/recipient age ratio (range)	0.14 (0.12–0.25)	0.66 (0.38–0.87)	1.04 (0.59–2.11)	**<0.001**
Recipient weight, kg (range)	8.8 (6.2–11.2)	8.8 (7.4–10.1)	12.5 (8.6–15.5)	0.112
Donor weight, kg (range)	5 (5–7)	10 (8–12)	14 (12–16)	**<0.001**
Donor/recipient weight ratio (range)	0.58 (0.49–1.05)	1.13 (0.86–1.42)	1.16 (0.75–1.82)	0.194
Recipient pretransplant z score, median (IQR)	–1.43 (–2.42 to 0.80)	–1.59 (-2.15 to –1.09)	–0.60 (–2.22 to –0.36)	0.259
ABO identical	3 (60%)	7 (78%)	19 (90%)	0.239
Type of graft				0.15
Isolated small intestine	0 (0%)	1 (11%)	6(29%)	
Liver-intestine-pancreas	3 (50%)	6 (68%)	12(57%)	
Multivisceral	1 (17%)	1 (11%)	1 (5%)	
Modified multivisceral	1 (17%)	1 (11%)	1 (5%)	
Liver-containing graft	4 (80%)	7 (78%)	14 (67%)	0.744
Reduced liver	1/4 (25%)	1/7 (14%)	7/14 (50%)	**0.04**
Colon-containing graft	0 (0%)	4 (44%)	3 (14%)	0.08
Cold ischemic time, min, median (IQR)	355 (315–487)	392 (360–465)	380 (355–451)	0.728
Abdominal closure				
Primary closure^*a*^	4 (80%)	8 (89%)	18 (86%)	0.901
Delayed primary closure	0 (0%)	3 (33%)	7 (33%)	0.311
Basiliximab induction	5 (100%)	5 (56%)	14 (66%)	0.219
Maintenance immunosuppression				0.239
Steroids + tacrolimus	5 (100%)	5 (55%)	12 (57%)	
Tacrolimus + steroids + antimetab.	0 (0%)	4 (44%)	6 (29%)	
Tacrolimus + steroids + rapamycin	0 (0%)	0 (0%)	3 (14%)	

Recipient, donor, operative, and immunosuppression characteristics of the EI, LI, and EC donors.

^*a*^Comparison with prosthetic closure. This proportion includes those who underwent delayed primary closure.

EC, early childhood; EI, early infant; IQR, interquartile range; LI, late infant; PN, parenteral nutrition.

An ISBtx was performed in 0, 1, and 6 patients in the EI, LI, and EC groups, respectively (Table 1). A similar proportion of each group received a liver-containing graft; however, a significantly higher proportion of recipients in the EC group received a reduced liver graft (Table 1). No patient in the EI group received a colon-containing graft. The ability to perform either primary or prosthetic closure of the abdomen was higher in the EI group, and no patient in this group required a second-stage closure (Table 1). The cold ischemic time (in minutes) was similar between the groups. Neither the induction nor the maintenance immunosuppression differs between groups.

Neither the length of intensive care nor hospital admission differed significantly between groups (Table 2). Postoperative PTLD was more common in the EI group; however, this difference was not statistically significant.Severe acute rejection of the small bowel was similar across the groups. The incidence of GVHD was higher in the EI group, but did not reach statistical significance (*P* = 0.206). In a similar manner, the incidence of significant immunological complications (severe acute rejection or GVHD) was higher in the EI group (EI: 4/5 [80%], LI: 5/9 [56%], EC: 7/21 [33%], *P = *0.134) but was not statistically significant.

**TABLE 2.** Outcome of study groups

	EI (≤6 mo) N = 5	LI (7–12 mo) N = 9	EC (13–36 mo) N = 21	*P*
ICU length of stay, d, median (IQR)	7 (4–26)	3 (2–5)	4 (1–16)	0.545
Hospital length of stay	60 (39–79)	69 (41–102)	52 (33–80)	0.564
Timing of postoperative enteral feed, d, median (IQR)	5 (3–8)	5 (3–10)	5 (3–10)	0.915
Postoperative day TPN ceased, median (IQR)	13 (13–18)	18 (15–23)	22 (18–37)	0.280
Severe small bowel rejection	2 (40%)	4 (44%)	6 (29%)	0.674
Postoperative PTLD	2 (40%)	1 (11%)	3 (14%)	0.334
GVHD	2 (40%)	1 (11%)	2 (10%)	0.206
Significant immune complication^*a*^	4 (80%)	5 (56%)	7(33%)	0.134
1 y graft survival	3/5 (60%)	6/9 (66%)	16/21 (76%)	0.721
3 y graft survival	0/5 (0%)	6/9 (66%)	15/20 (75%)	**0.012**
5 y graft survival	0/5 (0%)	4/9 (44%)	12/20 (60%)	**0.055**

^*a*^Significant immune complication defined as severe acute rejection or GVHD.

EC, early childhood; EI, early infant; GVHD, graft vs host disease; ICU, intensive care unit; IQR, interquartile range; LI, late infant; PTLD, posttransplant lymphoproliferative disorder; TPN, total parenteral nutrition.

**FIGURE 1.**
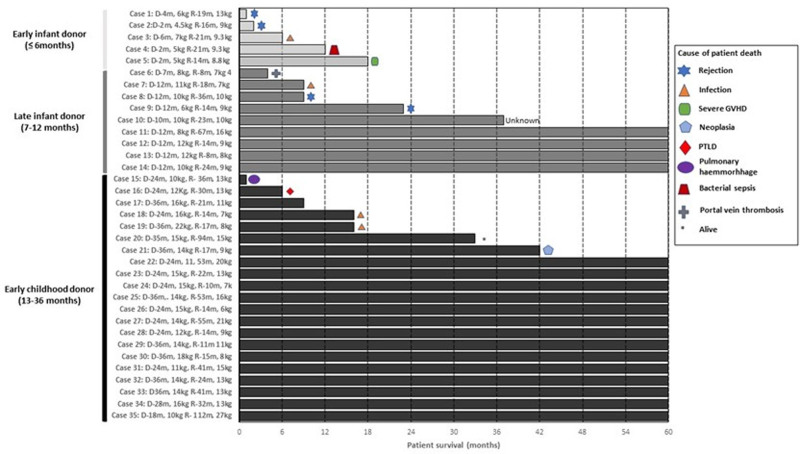
Patient survival. Bar graph depicting the survival of each patient based on donor age. PTLD, posttransplant lymphoproliferative disorder.

The duration of each recipient’s survival is graphically depicted in Figure 1, along with the cause of death. Significant immune complications were the cause of death in 3 of 5 patients (60%) in the EI group, with 2 children dying from rejection and the other from GVHD. Two patients in the EC group underwent late retransplantation at 46 and 53 mo. No children in the EI or LI group underwent retransplantation. The 12-mo graft survival did not differ significantly between groups (EI: 3/5 [60%], LI: 6/9 [66%], EC 16/21 [76%], *P = *0.721). The maximum postoperative survival in the EI group was 27 mo (Figure 1); therefore, survival at 3 and 5 y for the EI group was significantly lower than that of the LI and EC groups at both these time points (Table 2). The survival of the LI and EC did not differ.

## DISCUSSION

In the United Kingdom, there is a scarcity of intestinal grafts of suitable size for many pediatric patients on the waitlist.^13^ The appeal of using grafts from young donors is obvious, as the size of the graft from these donors is compatible for recipients with a less capacious abdominal domain. The findings of this study demonstrate that the outcomes of intestinal donors within the first 6 mo of life are inferior to those of older donors, as demonstrated by lower 3- and 5-y survival rates. Significant immune-mediated complications occurred in all but one of the recipients from a donor aged 6 mo, and younger and resulted in the death of 3 of 5. Although it did not attain statistical significance, the incidence and mortality rates of significant immune-mediated complications decreased progressively in the groups that received grafts from donors in LI and EC. The rate of severe acute rejection (23/35; 66%) in patients in this study is higher than that reported in series from other centers. Chang et al^8^ reported an acute rejection rate of 63%–67% in their series of pediatric intestinal recipients; however, it must be noted that this only included the initial 90 d from transplant. Tomoaki et al^14^ reported that 67% of pediatric recipients experienced biopsy-proven rejection, with the incidence of severe acute rejection being 20%, during a longer period of follow-up. Previous publications from our institution have also reported a higher rate of rejection (69%) in the first 6 mo of follow-up, when all donors, regardless of age, were included in the series.^15^ These observations may be explained by different protocols of surveillance or clinically indicated biopsies, immunosuppression regimes, and histological interpretation.

The outcomes of IT using grafts from very young donors, including neonates, have been reported previously in the form of case reports only.^16,17^ Larger case series have included recipients who received grafts from young infant donors; however, the outcomes of this subgroup have not been reported.^8,18-20^ Previous authors have reported technical complications to be a concern with grafts from small donors.^8^ However, our results indicate that inferior outcomes with grafts from young infants (6 mo and younger) appear to be mediated by infection or immunological complications rather than technical problems. In our series, a single child was lost as a result of a technical complication (portal vein thrombus). It is possible that a mechanism, yet to be described, could contribute to a more brittle immunological balance in recipients of intestine-containing grafts from young infants (younger than 6 mo). The potential causes could be a distinct population of donor-derived graft resident immune cells or an altered microbiota in very young donors. We acknowledge that the immunosuppressive regimen for recipients of EI donor grafts was less intense than that for LI and EC, and this may be in part responsible.

The small intestine contains the largest mass of immune cells and lymphoid tissue of all the solid organs transplanted.^1,21^ These characteristics contribute to the graft immunogenicity and the related complications seen in IT recipients, as compared with other solid organ transplant recipients.^21,22^ Age-related changes in the immune system are well documented and have clinical relevance for organ transplantation.^23,24^ Innate immune mechanisms are heavily relied upon in EI with maturation of the adaptive arm throughout childhood. After IT, the recipients’ immune cells repopulate the small bowel graft, but the enteric epithelium retains the donor genotype and is therefore highly immunogenic.^1^ Shortly after transplant, recipient antigen-presenting cells infiltrate the graft and comprise 75% of this population within 10 d.^25^ Furthermore, these persisting donor cell subtypes are known to be potent secretors of cytotoxic cytokines such as perforin and Granzyme-B.^26^ The more antigen-naive mucosal and immune cells within a young infant donor’s small intestine may interact with the recipients’ infiltrating cells in a different manner than that of older donors, and result in more severe and refractory rejection or GVHD. The clinical management becomes even more complex as responding with excessive suppression of the immune system can lead to infection or malignant transformation of the immune cells (PTLD).^27^

The intestinal microbiota of infants is known to differ from that of older children and adults.^28^ Flora consistent with that from the birth canal are the first detected in the intestine of an infant, with changes occurring after the introduction of breast milk, formula, and solid food.^28^ The intestinal microbiota diversity increases over time, and by 1 y of age, it is similar to that of an adult.^28,29^ In a manner similar to Crohn’s disease, the commensal bacteria of the transplanted intestine are thought to interact with the immune cells in the epithelium to elicit allograft rejection.^30^ The more restricted microbiota of young infant donors is a potential cause of an excessive immune response from older recipients through several mechanisms. In addition, Mathew et al^21^ demonstrated that graft-infiltrating lymphocytes isolated from rejected small intestinal allografts proliferated in response to bacterial stimulation, whereas those from grafts without rejection did not. These authors concluded that immune cell sensitization to the intestinal microbiota contributes to allograft injury.^21^ Conversely, several microbes, such as those from the *Clostridium* genus, have been shown to induce FoxP3 regulatory T cells within the lamina propria, which are known to have a tolerogenic effect.^28^ A lower proportion of regulatory T cells within the young infants’ graft after transplantation may explain the increased immunogenicity.

A limitation of this study is the small sample size and its retrospective nature, a problem inherent with IT research due to its infrequent requirement and performance at a select number of centers. Variables such as an increased incidence of recipient prematurity and nonanatomical causes of intestinal failure (dysmotility and mucosal disorders) in the younger recipient groups (EI and LI) are examples of variables that may confound the observations. We acknowledge that our study has not investigated the mechanistic basis of our observation and merely reflects our suspicions and hypotheses. Given the insignificant differences in immune complications, it is possible that factors (either donor or recipient) that we have not analyzed are influencing the poorer survival of recipients of grafts from donors aged younger than 12 mo. A benefit of single-center research is the consistency of the approach to the assessment and management of these complex children, both before and after transplant. We acknowledge that our study has not investigated the mechanistic basis of our observation, and merely reflects our suspicions and hypotheses. Given the insignificant differences in immune complications, it is possible that factors (either donor or recipient) that we have not analyzed are influencing the poorer survival of recipients of grafts from donors aged younger than 12 mo.

In conclusion, we recommend caution with the use of grafts from very young infants due to the high rate of early patient death from immune-mediated sequelae. The mechanism of this observation is unclear at present and should be a focus of future research.
